# Supplementary material for: Are carbon emissions trading and green financial instruments synergistic? -Comprehensive quantitative research based on content analysis
Source: PLoS One. 2024 Mar 7;19(3):e0298601. doi: 10.1371/journal.pone.0298601 (PMC10919601; doi:10.1371/journal.pone.0298601)
Supplement: S1 Data — (ZIP) [file pone.0298601.s001.zip › Data/地级市Z_F工作报告.zip!/┴╔─■/╡ñ╢1⁄2╩╨/└┤╫╘╣1⁄2╓┌║┼í╛┬φ┐╦╩2╛▌═°í┐_2009.docx]

各位代表：

现在，我代表市人民政府向大会报告工作，请予审议，并请市政协委员和其他列席人员提出意见和建议。

一、2008年政府工作的回顾

2008年，按照市委提出的超常规、跨越式发展总体要求，市政府组织和带领全市人民，抢抓机遇，应对挑战，顽强拼搏，开拓奋进，在宏观形势趋紧的情况下，圆满完成了市十四届人大一次会议确定的各项任务。

——国民经济总量快速增长，综合实力迈上新台阶。2008年，预计实现地区生产总值550亿元，比上年增长16.5%;规模工业增加值完成153亿元，增长25%;全社会固定资产投资完成350亿元，增长50%；财政一般预算收入完成37.5亿元，增长30%；社会消费品零售总额完成198亿元，增长22.3%；实际利用外资完成3亿美元，增长104%；外贸出口总值完成13.5亿美元，按可比口径增长15.5%；城镇居民人均可支配收入达到11641元，增长18.6%；农民人均纯收入达到6580元，增长18.7%。

——工业经济增势强劲，质量和效益不断提高。工业经济在受到国际金融危机冲击的情况下，仍然保持较快增长，预计工业主业收入增长36%，实现税金增长26%，实现利润增长15%。新增规模企业204户，总数达到1087户。风机法兰盘、不锈钢冷轧板、船舶配套工业园、引水工程钢管、东北亚纺织服装基地、手表工业园等工业大项目进展顺利，600万吨修造船基地项目已开工实施。70个重点工业项目中，凤辉矿业硼铁分离等28个项目已经实现全部或部分竣工投产；南洋制衣等23个新开工项目实际开工率已达70%；10个重点前期项目正在积极推进。预计实现高新技术产品增加值40.5亿元，增长60%。企业研发能力不断增强，9户企业被认定为省级企业技术中心。手表公司等国有企业成功改制。装备制造业、农产品加工业等主导产业和优势产业发展速度加快，十大重点产业集群逐步启动。努力破解民营企业融资难题，通过担保公司共为中小企业新增贷款4亿元。

——农业产业结构不断优化，农业和农村经济稳步增长。克服诸多不利因素影响，农林牧渔各业全面丰收。粮食产量达到91.5万吨，是历史上第三个丰收年。种植业新增设施面积4.7万亩，新建畜禽标准化养殖小区134个，海水精品、淡水珍品渔业带规模继续扩大，水产品总产量达到53万吨。新增规模以上农产品加工龙头企业25户，总数达到190户。实现农产品出口创汇4.6亿美元，增长15％，稳居全省第二位。动植物疫病得到有效防控，新增安全农产品生产面积30万亩，无公害、绿色、有机食品认证28个。完成8座水库除险加固和45项小型灌溉水源工程，鸭绿江防洪护岸工程中两项回水堤工程全面启动。集体林权制度改革已完成应改面积的97.5%。植树造林15万亩。新建农村沼气用户3000户，完成秸秆气化站一期工程25处。解决了9.2万人饮水安全问题。落实党的惠农政策，发放农林牧渔及水利等各类补贴资金5.48亿元。大力推进新农村建设，为75个示范村编制了村屯整治建设规划，引领示范村新增项目115项，投入资金3.63亿元。积极扶持发展农民专业合作社，总数达到308个。出台了支持县域经济发展政策及措施，搭建了银企合作平台，加快了县域经济产业园区建设步伐。

——旅游业健康发展，消费市场繁荣活跃。以鸭绿江品牌为核心的旅游品牌打造及五条精品线路推介取得明显成效，虎山长城景区改造基本完成，阿里郎旅游休闲乐园等项目积极推进。成功举办了鸭绿江旅游节、辽宁台湾周等节庆活动，丹东旅游形象广告进入国内主流媒体播出。与国内外近千家旅行社签订互送团队人数62万人次，黄金周和假日旅游取得明显成效。完成了丹东旅游规划编制工作。全年预计接待国内外游客人数1449万人次，增长29%。旅游业总收入104亿元，增长29%。其中，旅游外汇收入7410万美元，增长25%。大商新玛特等一批商业重点项目顺利实施，首家五星级酒店正式开业，交通物流中心等一批企业初具现代物流企业雏形。继续实施“双进社区”工程和“万村千乡市场工程”，农村四级流通网络基本形成，农商对接、节假日联展联销等活动进一步活跃，市场经济秩序保持稳定。

——招商引资屡创新高，对外开放步伐加快。围绕城市新区、十大产业集群和各类工业园的开发建设，组织500个招商团组共1600多人，赴日、韩、港及长三角、珠三角地区开展大规模招商活动，共签订合作项目107项，协议资金约340亿元，合同外资额约40亿美元。洽谈、签约、到位资金和开工项目在规模、质量上均创历年最好水平，一批重大项目得以引进并实施。预计全年到位域外资金120亿元，比上年增长58.7%。努力克服外贸出口不利形势影响，帮助出口企业解决各种难题和搭建出口平台，外贸进出口保持增长。积极推进境外投资和工程承包，全市完成外派劳务6600人。

——固定资产投资快速增长，城市基础设施日臻完善。丹东境内投资34亿元的东北东部铁路项目即将开工建设；总投资400亿元的丹东核电站项目已签署框架协议；总投资35亿元的金山热电项目正在组织资金和动迁工作；总投资48亿元的华能丹东电厂二期工程已正式上报国家发改委核准；丹东境内投资95亿元的丹通高速公路、投资8.67亿元的丹海高速公路已开工建设；投资6.2亿元的丹东至五龙背、北外环、浪东线三条快速通道进展顺利；总投资4.2亿元的丹东客运站搬迁扩建工程正在加快动迁；丹东港扩建6个主要泊位正在施工，主航道、西南航道等正在实施疏浚工程，全年完成投资26亿元，全市港口吞吐量达到3450万吨，增长25%；投资8.5亿元，实施填海造地9平方公里；丹东火车站站舍楼改造工程已达到简易交付使用条件；丹东机场改扩建项目正在积极推进；鸭绿江西水道整治工程进展顺利；总投资18.4亿元的三湾水利枢纽及输水工程前期准备工程已开工建设；蒲石河抽水蓄能电站已累计完成投资19亿元；一期投资7亿元的500千伏输变电工程已完成投资3亿元；总投资6亿元的新建、扩建220千伏输变电工程已竣工投入运行。丹东新区控详规、国门湾城市设计和专项工业园控详规初步完成，新区固定资产投资完成60亿元，增长200%。5平方公里土地实现了“五通一平”，达到企业入驻条件，新引进千万元以上项目45个，总投资225亿元，新开工项目32项，竣工项目10项，其中牛顿方式新城区开发、恒和会展中心、成功花园大酒店、新加坡城、曙光集团SUV乘用车、市民服务中心和新建二中等项目进展良好，大豆深加工项目一期工程已基本完工。新区基础设施建设快速推进，形成了四纵四横的主干网道路，相应的配套设施和绿化、亮化初具形象。

——财政收支结构趋于合理，金融保持平稳运行。努力克服财政减收增支的不利因素，加强税费征管，加大税费清欠力度，确保各项税费及时足额入库，财政收入实现高速增长。预计全地区全口径财政收入达到78.4亿元,增长19.1%。财政支出结构进一步优化，财政各项惠民政策得到落实。全年一般预算支出完成75.7亿元，增长25.4%。金融运行平稳，全市银行业机构存贷款总额大幅度增加，贷款不良比例持续下降，信用环境进一步优化。商业银行增资扩股2.55亿元，达到现代商业银行管理标准。

——民生工程富有成效，人民生活逐步改善。对城乡群众承诺的13件实事全面展开，其中，城区公园和内河整治系列工程顺利实施。金山供水工程已经完工。垃圾处理、污水处理、东坎子燃气等工程全面启动。38片5万平方米以下棚户区改造任务积极推进，实施了历史上最大规模的旧小区改造，127个旧小区得到治理，市容综合整治取得明显成效。完成了一号干线贯通、街巷路改造等工程，二号干线续建工程曙光路以西段通车。全地区实现就业6.72万人，实现了零就业家庭每户至少1人就业的目标，城镇登记失业率为4.62%。全面启动了城镇职工生育保险和城镇居民基本医疗保险，社会保障体系不断完善，养老等社会保险参保人数有较大增加。提高了城市居民最低生活保障标准，城市低保户和低保边缘户取暖问题得到解决。投资3890万元，购建廉租住房2.2万平方米，解决了432户特殊困难家庭住房问题。改造建设农村低保户危房1200多户。兴边富民行动取得较大成效。

——社会事业全面发展，精神文明建设水平显著提高。东港市、振兴区率先实现高水平、高质量普九工作目标，农村中小学“现代远程教育工程”全面完成。重点改造农村中小学危房73所3.3万平方米，建设农村九年一贯制学校10所，新四中正式投入使用。人才和引智工作有了新突破。“新农合”参合率达到90%以上。城市社区卫生服务基本实现了全覆盖，社区卫生服务中心全部纳入医保定点范围。开展“美丽丹东”特色系列文化活动，宣传了丹东城市名片。完成了20户以上自然村“村村通”广播电视任务，元宝山广播电视观光塔主体工程基本完工，抗美援朝纪念馆免费开放。竞技体育水平和实力得到大幅提升。低生育水平继续保持稳定。公民特别是未成年人思想道德建设得到加强，群众性精神文明建设创建活动取得新进展。军民军政关系进一步巩固，全民国防教育和民兵预备役工作得到加强。开展了有史以来我市规模最大的汶川特大地震救灾捐助活动，共捐献款物合计1.2亿元。

——民主法制全面推进，和谐丹东建设进展顺利。坚持向市人大及其常委会报告工作和向市政协通报情况制度，自觉接受监督，认真听取民主党派、工商联、无党派代表人士和各人民团体的意见，共办理人大代表建议261件和政协委员提案392件，办结率和满意率均达到100 %。加大信访突出问题和疑难案件的排查调处力度，妥善化解各种矛盾，有效预防和控制了重大群体性事件发生，实现了奥运期间确保安全稳定的工作目标。扎实推进“五五”普法。全面推进依法行政，政府机关依法行政意识和市民法律意识不断增强。不断完善社会治安动态防控体系，全力加强社会治安综合治理等各项公安行政管理，严厉打击各类犯罪活动，有力维护了社会和谐和政治稳定。积极开展“环境年”建设活动，制定并实施了加强软环境建设一系列规定和措施；加强效能、执法和廉政监察，促进了机关作风建设。抓好源头治理，加大案件查处力度，廉政建设和反腐败斗争取得明显效果。高度重视安全生产工作，加大交通、消防等公共安全管理和整治力度，加强食品药品安全监管，健全和完善安全生产长效机制，有效预防和减少了各类安全事故发生，全市安全生产状况总体平稳。

外事、侨务、口岸、台务、民族、宗教、统计、保密、档案、接待、地方志、人防、气象、防震减灾等工作也取得了新成绩。

上述成绩的取得是在省委、省政府的正确领导和大力支持下，我们面对国际金融危机，应对重大挑战和考验，带领全市人民同心同德、顽强拼搏的结果，成绩来之不易。在此，我代表市政府向全市工人、农民、知识分子，向各民主党派、工商联、无党派代表人士和社会团体，向驻丹部队和武警官兵、中省直驻丹单位，向所有关心、支持丹东发展的海内外朋友表示衷心的感谢并致以崇高的敬意！

在肯定成绩的同时，我们也要清醒地认识到，全市经济和社会发展还存在一些突出问题。一是国际金融危机对经济增长造成了一定影响。主要经济指标增长呈现先扬后抑的态势，出口受到前所未有的影响，部分工业企业处于停产、半停产状态，极大地制约了我市经济的快速增长。二是项目结构还需进一步优化。虽然采取了一系列招商引资超常规的手段和措施，并取得了较大成果，但项目建设中大项目少，尤其是工业大项目少的局面仍然没有得到根本改变。三是企业融资受到制约。受年初宏观调控政策影响，贷款遇到困难，下半年应对金融危机和宽松货币政策措施落实效果不明显，加之担保公司等金融服务业发展缓慢，难以形成对中小企业的有效支持。四是市本级财政统筹乏力。一方面停产、半停产企业对我市税收影响较大，导致财政增长乏力，市本级财政占全市财政总量的比重呈逐年下降趋势。另一方面财政增支因素不断增多，财政增长速度难以跟上刚性支出增长速度，导致财政运行尤其是市本级财政运行步履维艰。五是民生工作还需进一步加大力度。由于历史欠账多，财政支持能力有限，导致一些民生工程进展较慢，涉及百姓住房、社会保障等方面的一些遗留问题没有得到彻底解决。六是机关作风建设还需加强。机关办事效率不高、工作思路不宽、缺乏创新的状况没有彻底改观，一些干部安于现状、不思进取、推诿扯皮等现象仍然存在。这些问题已引起我们的高度重视，并将采取强有力措施切实加以解决。

二、2009年经济和社会发展的主要任务

当前，国际金融危机的冲击和影响仍有加剧的趋势，使我市外贸出口、企业生产乃至经济运行面临着巨大考验，这也是丹东遇到的前所未有的挑战。困难之中必然蕴含机遇。我们遇到的困难大，面临的机遇更大。国家拉动内需，在基础设施、民生工程等建设上扩大投资，使丹东一批重点项目实现重大突破，形成了实现跨越发展强有力的增长点；南方沿海地区产业、企业转移，使我们在吸引资金、项目上得到了更多的机会；辽宁沿海经济带上升为国家战略，为丹东发展带来了重大契机。更重要的是，通过新一轮思想大解放，丹东经济发展的氛围空前高涨，全市干部群众上下同心，盼发展、快发展的心情更加迫切，信心更加坚定。所以从当前总的形势看，中央保持国民经济平稳较快发展的决心没有变，全省经济发展的基本态势没有变，我市实现经济跨越式发展的目标没有变，只要我们变压力为动力，化挑战为机遇，抢抓机遇，迎难而上，团结一致，奋力拼搏，丹东就会实现大发展、快发展。为此，我们确定2009年全市经济和社会发展的指导思想是：高举中国特色社会主义伟大旗帜，以党的十七大、十七届三中全会和中央经济工作会议精神为指导，深入贯彻落实科学发展观，按照省委、市委十届七次全会的要求和总体部署，紧紧抓住东北老工业基地全面振兴和辽宁沿海经济带战略的双重机遇，以促进经济平稳较快增长为主线，以国家拉动内需为契机，以改革开放为动力，以新城区建设为重点，以丹东东港同城化为载体，以招商引资为牵动，以狠抓项目为核心，以提升地区经济实力为目标，以建设社会主义核心价值体系为保障，以解决人民群众实际问题为落脚点，大力实施“落实年”活动，积极应对挑战，坚定发展信心，努力实现丹东经济社会又好又快发展。

2009年全市经济和社会发展的预期目标是：地区生产总值增长16%；规模以上工业增加值增长23%；全社会固定资产投资增长40%；全社会消费品零售总额增长18%；外贸出口总值增长18%；实际利用外资增长33%；地方财政一般预算收入增长20 %；城镇居民人均可支配收入和农民人均纯收入分别增长16%和13 %。为全面完成上述预期目标，要全力抓好以下重点工作：

（一）以重大基础设施建设拉动为重点，在促进经济增长上实现新突破

紧紧抓住国家扩大内需、促进经济平稳较快增长的有利时机，认真研究掌握国家产业政策、发展方向和世界金融危机的动态，积极推进重大基础设施建设项目实施。全市力争实施千万元以上建设项目300个，投资总额820亿元，重点调度项目115个，计划完成投资180亿元。加快一批重大在建项目建设进度：蒲石河抽水蓄能电站完成投资9亿元，进行设备安装；东北东部铁路进行路基、隧道等工程建设；丹通、丹海高速公路主体工程进入全面施工阶段；500千伏输变电一期工程年底竣工投入运行；鸭绿江西水道整治工程年底基本完工；丹东港西南航道工程争取完工；污水、垃圾处理厂年底建成投入使用。确保一批重大项目开工建设：积极协助铁道部落实总投资228亿元的沈丹铁路客运专线建设条件，确保6月份开工建设；全力配合铁三院和省发改委做好丹大铁路客运专线各项前期准备工作，力保今年开工建设；华能丹东电厂二期工程争取下半年开工建设；金山热电集中供热工程争取上半年开工建设；计划投资32亿元的丹东港扩建工程，完成原2-5号泊位改造工程，开工建设外1-4号泊位；丹东公路客运站搬迁扩建工程，年初开工建设，年底前完成主体工程；总投资2.5亿元的丹东机场改扩建工程加快完成项目核准等审批手续，下半年开工建设；三湾水利枢纽及输水项目主体工程上半年全面开工。积极推进一批重大项目前期工作：丹东核电站项目，完成项目选址、可研等前期工作；总投资12.6亿元的鸭绿江公路大桥项目，做好开工前的各项准备工作。相关部门要建立大项目专门协调机构，做好大项目的各项服务工作，确保大项目顺利实施。

（二）以加快实施丹东东港同城化和新区建设为重点，在打造北黄海经济带上实现新突破

加快打造北黄海经济带。积极推进“西进东联”步伐，向西以辽宁沿海经济带开发建设为依托，主动接受大连的辐射。向东以打造东北东部地区最便捷的出海大通道为主线，积极加强与东北东部13个市的区域经济合作，最大限度地发挥我市在东北东部地区对外开放窗口作用，增强我市在区域经济发展中的重要影响力。

加快丹东东港同城化步伐。抓紧编制同城化发展的中长期规划，重点是产业发展、土地利用等专项规划。高标准改造201国道，实现全程亮化；加大浪东线建设力度，形成城际快速干道；积极推进城际轨道交通建设前期工作；加快西水道整治工程进度，确保水路畅通；实施20万吨供水工程，解决新区、临港产业园区及东港用水问题。抓紧研究城际大公交机制，实现城际客运公交化运营；加快市民在社会保障、就业等方面享受同城待遇。加强前阳镇的城镇规划和产业规划，发挥前阳开发区在同城化中的节点作用。

加快丹东新区建设。推进黄海大道、中心路等11条道路和国门湾护岸工程、新区绿化亮化美化工程、通讯电力工程等基础设施建设，加快金融大厦、体育馆、图书馆、综合医院、国门广场等公用设施以及恒和会展中心、成功花园大酒店、新加坡城、香港美食街、高档商住楼和65万平方米公寓楼等商业地产项目建设，促进SK保税物流园、不锈钢交易市场、建材装饰城等商贸物流项目尽快落地，力争阿里郎旅游休闲乐园、安民山主题公园等休闲娱乐项目开工建设，确保二中新校区投入使用，市民服务中心主体建成，形成较为完善的城市功能。启动合作区商贸旅游区改造和浪头老城区改造工程。

推进临港产业园区产业集聚。新整理土地9平方公里，规划建设装备制造园、新加坡工业园、手表工业园、不锈钢工业园、钢铁工业园等园区，实现同类项目产业集聚。重点加快风机法兰盘生产、引水工程钢管制造、大无缝铜拉杆生产、静脉工业园、标准厂房等重点在建项目建设进度，积极推进不锈钢冷轧板、曲轴加工制造、测控装备、风电机组叶片制造、游艇制造、不锈钢加工物流园等大项目及早开工，确保SUV乘用车等项目完成主体工程，以项目连片推进港城一体化发展。

（三）以招商引资为重点，在对内对外开放上实现新突破

拓宽招商领域，提高内外资利用水平。以珠三角、长三角、东北东部、香港、澳门、台湾和日本、韩国、新加坡等地区和国家为招商重点，以新区开发、老区改造、十个产业集群建设为招商载体，谋划包装一批高质量项目。创新招商方式，充分发挥专业招商分队和团组的作用，采取多种招商方式，实现招商引资新突破。争取实际利用外资4亿美元，增长33%，引进内资180亿元，增长50%，签约亿元以上项目110个，与世界500强企业对接洽谈项目2-3项，同时要特别注重加强与国内500强企业的对接洽谈。创新理念，办好台湾周活动，打造对台合作平台。

创新项目工作机制，扩大项目储备。建立项目落实、运行、推进工作机制，实行项目全过程跟踪服务，确保重点项目早开工、早投产、早见效。加强对重大项目的单体包装和中小项目产业集群的整体包装，市级项目库动态库存达到800个，重点推进项目达到120个。紧紧抓住国家投资向农村基础设施建设倾斜的有利时机，加大项目包装和前期工作力度，争取国家更多的资金支持。建立政府与投资者的双向约束机制，确保项目已签约的尽快注册，已注册的尽快开工，已开工的加快建设，已竣工的尽快投产达效。

改善外贸、外经增长方式，扩大对外交流渠道。加大对出口企业的扶持力度，重点支持名牌产品、高新技术产品、高附加值产品和地方产品出口。精心组织重要交易会、展会的企业参展工作。充分利用国家“提升沿边开放”政策，争取出口加工区尽快通过国家审批，做好中朝边境综合保税区的前期工作。巩固拓展与国外友好城市关系。加快开通丹东口岸机场国际航线。

（四）以支持企业积极应对金融危机为重点，在提升工业经济实力上实现新突破

采取有力措施，解决生产经营中的各种困难。集中力量帮助现有企业解决生产经营中遇到的困难和问题，研究、制定扶持政策，增强企业克服困难的能力和信心。以支持企业降本增效为重点，着力协调解决好煤、电、油、运等生产要素供应，保证企业生产正常运行。市县乡都要派专人进驻企业，协调企业妥善处理裁员与放假问题。同时，要充分利用金融危机造成原材料价格走低的时机，制定引导企业加大投入的导向性政策，促进企业改造，增强企业信心和竞争力。重点支持曙光汽车、五兴化纤、五一八等龙头企业发展，使其充分发挥潜能。全力启动停产、半停产企业，盘活存量资产，优化增量资产，培养恢复性经济增长点。认真分析解决项目建设中存在的问题，帮助一批已经竣工的重点项目尽快投产。引导企业加强内部管理，调整产品结构，提高产品质量，增强抵御风险能力，努力帮助企业开拓国际国内市场。大力扶持中小企业发展，通过建立担保机构风险补偿机制，协调加大信用担保机构对中小企业的支持力度，启动市县两级财政担保体系，定期组织银企对接，有效缓解中小企业融资困难。全力做好省中小企业集合债发行工作，推动中小企业直接融资，力争年内实现发债8亿元。积极引导民营资本投资融资服务业，加快建立村镇银行，提高中小企业贷款担保公司能力，形成16亿元的担保能力。确保规模以上企业全部参加省公共征信体系建设工程，提升企业融资信用等级。加快企业上市融资步伐，帮助辽宁欣泰输变电等企业做好上市前期工作。跟踪落实已出台的扶持政策，帮助房地产业渡过难关。

狠抓工业大项目，做大做强主导产业和骨干企业。利用好国家扩大内需政策和措施，积极谋划和推进一批工业项目。实施投资额超亿元工业项目50个。对100万吨硼钢、200万吨大豆深加工等在建项目要加快实施进度，尽快竣工投产；对五一八大型曲轴和压力机改造等新开工项目要尽快开工建设；对16万吨铜版纸等已经竣工但尚未投产达产的项目要促其尽快投产达产。同时，围绕主导、优势产业集群和国家重点支持的产业包装项目，新包装超亿元项目50个以上。突出抓好产业优化升级，装备制造业实现主业收入达到260亿元、农产品加工业达到200亿元、矿产品加工业达到190亿元、电力工业达到21.6亿元。做大做强骨干企业，新增超亿元企业10户，超5亿元企业10户，超10亿元企业4户，力争超百亿元企业1户。大力推进工业园区建设和产业集群发展，新建标准厂房100万平方米以上，新入驻企业达到100户以上。

加强科技研发体系建设，提高自主创新能力。加大科技投入，加强企业研发体系建设、科技企业孵化器建设和科技型中小企业培育。加速推进无水印染产业化基地建设。积极推进曙光集团城市客车、五一八船用曲轴等一批高附加值、高技术含量产品的试成投产，加强节能减排新技术、新产品的研发及产业化推进。加快技术中心建设，增强企业自主研发能力。强化产学研合作和对外科技合作，为企业提供技术支持和技术服务。

（五）以园区建设和特色农业为重点，在县域经济发展上实现新突破

引进产业集群，打造特色产业集聚基地。充分利用资源，加强产业化龙头企业建设，抓好龙头企业与产业链条的衔接，形成以龙头企业为骨干的农产品加工产业集群，重点抓好百万头生猪、亿只肉食鸡屠宰及其它养殖项目的发展。明确重点发展产业集群目标，实现从招企业向招产业转变。东港农副产品加工产业集群，凤城硼铁、黄金产业集群，宽甸硼、石材加工产业集群要见形象、上规模。加快东港开发区、凤城现代产业园区和宽甸城南园区等省级县域重点园区的基础设施建设，促其尽快形成产业集聚基地。努力为县域经济发展提供土地、融资、标准化厂房等支持，促进项目早日落地、投产见效。统一各产业园区内的优惠政策，实现招商资源的合理配置和共享。

加快县城改造进程，着力提升县城建设水平。抓好县域规划和基础设施建设，把县城建设同房地产开发、服务业发展统筹考虑，坚持县城改造、园区开发和小城镇建设齐头并进，全面启动县城的房地产业，大力发展现代服务业，完善城市功能，使县城规模逐步扩大，品位不断提升，聚集力和辐射力明显增强。全年县域经济各项指标要确保不低于两位数增长。把县域经济发展与促进农民增收、加快城镇化建设相结合，通过县域经济的快速发展，实现农业向“企业化、产业化”转变，推动我市新农村建设。

狠抓农业现代化，大力发展特色产业。稳定粮食生产，突出发展特色产业，新增设施农业小区1.5万亩，新建标准化畜禽养殖小区124个，实现水产品总产量59万吨。新增规模以上农产品加工企业30家，总数达到220家。完善农业科技创新推广示范体系，推介农业新品种、新技术50项。加强农产品质量标准、检验检测、质量认证和执法体系建设，推进农业保险，新增安全农产品生产面积25万亩，无公害、绿色、有机“三品”质量认证20个。

认真落实党的“三农”政策，改善农民生产生活环境。认真落实农村土地承包和各项强农惠农政策，积极推进农村土地、集体林权制度以及农村各项配套改革。对东港四个农垦企业进行资源整合，打造现代农业园区。积极引导、扶持和规范农民专业合作社发展，年内总数达到500家。继续实施东港大型灌区改造项目。组织实施13项小型农田水利工程，完成11座病险水库除险加固和18万亩水土流失治理任务，解决10万人的饮水安全问题。大力加强国家重点公益林、天然林、海防林建设及滨海大道绿化工程建设，完成植树造林11.5万亩。推进农村新能源建设，抓好农村沼气国债项目建设，启动农村秸秆气化集中供气二期工程建设，新建农村沼气模式2000户，推广新型节能住宅1000户。拓宽农民增收渠道，减轻农民负担，提高农民收入。

（六）以旅游业为重点，在现代服务业发展上实现新突破

以旅游业为龙头，全面带动现代服务业蓬勃发展。大力实施构建大旅游战略，把旅游业作为主导产业加以培育，突显旅游业对丹东经济的拉动作用。加快鸭绿江精品旅游带、著名景点旅游线路和赴朝旅游产品的包装和推介，抓好北国之春、国际游客服务中心、大梨树影视城、虎山影视城等项目建设，加快建设东北东部影视基地。整合旅游资源，不断壮大观光旅游，大力发展休闲旅游，积极鼓励生态旅游，努力培育会议旅游，早日开通赴朝旅游。加大旅游宣传力度，举办好鸭绿江国际旅游节等各种节庆活动，继续在国内主流媒体开展旅游主题宣传，提高旅游服务水平，扩大丹东旅游影响力和知名度。努力把丹东打造成东北地区游客的聚集地、国内外游客的休闲地和赴朝旅游的集散地。

以提升传统服务业为支撑，加快培育新兴服务业。加快推进农副产品配送中心等一批商业重点项目建设，提升改造七经街、新安步行街，培育宝山女人街等区域商业中心，扶持新玛特等大型商贸企业做大做强。以“万村千乡”和“双进社区”活动为载体，积极开展以“促消费，保增长”为主题的农商对接系列活动，畅通城乡流通网络。加快“新网工程”建设，村级超市数量要覆盖全市75%的行政村。积极发展中介、金融保险、信息等新型现代服务业态，大力发展融资、债券、评估、担保等中介服务组织，不断提高中介服务的质量和竞争力。进一步整顿和规范市场经济秩序，严厉打击假冒伪劣行为，确保物价稳定和流通顺畅。

以港口和新建鸭绿江公路大桥为依托，助推现代物流业发展。积极争取新鸭绿江公路口岸区项目，建设综合服务区、货物存放和仓储集装箱场站等一流的服务设施。加快粮贸物流、烟叶物流中心、能源储藏基地等新建项目的开工，推进益享林集团物流、星源船舶燃料物流等续建项目的实施，带动发展港口运输、集装箱中转等相关物流服务企业，构建由综合物流园区、专业物流中心和现代物流企业组成的现代综合物流园区，努力把丹东打造成为东北东部区域性物流中心。

（七）以完善城市功能为重点，在改善群众居住环境上实现新突破

完善城市规划，抓好城市建设重点工程。修编城市总体规划，完善丹东新城区规划。完成五龙背镇和金山镇等重点区域及项目的规划编制工作，全市小城镇规划完成率达到100%。做好土地利用总体规划修编工作。完成二号干线续建工程，适时启动打通三号干线东尧段、花园东路改造等工程。抓紧实施东坎子煤气普及工程，年底前解决该地区10万户左右居民的供气问题。积极推进城市水源改造工程，解决金山镇、五龙背镇供水问题。开工建设客运站地下人防工程，搞好公共交通场站建设。

加强城市管理，改善群众居住环境。坚持集中整治与专项整治相结合，加强对市容和城乡结合部环境的综合整治及道路交通等专项整治，解决违法占道经营、交通秩序混乱、噪声扰民、城市造脏、私搭乱建、乱排乱放等问题，继续实施城市绿化、亮化工程，推进城市生态环境建设。深入开展打击违法排污、保障群众健康环保专项行动和环保攻坚惠民活动，完成污染减排工作目标。在主城区和公园新建10座水冲公厕，逐步解决群众如厕难问题。注重和加大对环境的保护力度，大力发展循环经济，实现节约发展、清洁发展和安全发展。全面完成城市旧住宅小区治理改造任务，积极推进5万平方米以下棚户区改造工程及改造后的小区道路、环境建设。高度重视居民回迁工作，妥善解决回迁过程中遇到的难题。抓好低收入困难家庭住房保障，对符合保障条件的低收入住房困难家庭实行实物配租和租赁住房补贴。加快推进村镇建设，实现第一批12个省级村庄整治试点村全部进入省级示范村行列。抓好供热监管和体制改革，继续推进集中供热。

（八）以壮大财源和强化金融服务为重点，在支持地区经济发展上实现新突破

统筹政府财力资源，确保公共服务有效供给。完善税源监控体系，强化税收征收管理，确保各项税收收入应收尽收和及时足额入库。规范非税收入管理，切实加强对财税的管理和统筹力度。加强社保基金征缴，有效整合、充分利用行政事业单位资产，整合政府财力资源。

优化支出结构，完善公共财政运行机制。树立过紧日子的思想，厉行节约，大力压缩会议、接待、公务用车等一般性支出，重点向“三农”、民生等方面倾斜。继续深化部门预算、国库集中支付、政府采购和“收支两条线”等改革，有效降低行政运行成本，加快法治财政、阳光财政和绩效财政建设。完善市以下财政管理体制，努力实现基本公共服务均等化。

优化金融生态环境，扩大对地方经济的支持力。完善金融工作协调机制，改善金融生态环境，提升地区金融竞争力和资金吸引力。引导金融机构着力调整优化信贷结构，加大对重点领域的信贷投放力度。鼓励金融机构产品创新，扩大有效担保范围，提高金融服务水平。推进信用和诚信体系建设，严厉打击恶意逃废债和失信行为，做好不良贷款清收工作。

（九）以切实解决城乡民生问题为重点，在构建和谐社会上实现新突破

切实抓好就业工作。充分发挥当前国家扩大投资和项目建设带动就业的作用，实现经济增长与扩大就业良性互动。实施积极的就业政策，鼓励支持大中专毕业生、失业人员、农民工等不同群体以自主创业带动就业。加大中小企业和劳动密集型企业政策扶持力度，千方百计增加就业岗位，严格控制企业的规模裁员。建立完善小额担保贷款、创业补贴、创业培训、创业孵化基地等就业扶持体系，全面落实普惠制就业培训、公益性岗位补贴、兜底安置等就业援助政策，建立就业援助长效机制，确保零就业家庭中有劳动能力和就业需求的人员实现再就业，困难家庭大学生保证其登记当年实现就业，全年实现实名制就业5万人，城镇登记失业率控制在4.6%以内。

切实抓好社会保障工作。积极推进各项社会保险扩面工作，力争将全地区城镇户口人员全部纳入城镇基本养老保险范围，基本实现全民养老保险，年内养老保险扩面2万人；实现失地农民养老保障制度与城镇职工养老保险制度并轨；启动工伤医疗保险；城镇职工基本医疗保险扩面2万人；城镇居民基本医疗保险扩面6万人；工伤保险扩面4000人；生育保险扩面5.5万人。

继续抓好为民办实事工作。在继续抓好去年为群众承诺的13件实事的基础上，再为城乡群众办15件实事：推进农村人畜饮水安全工程，新建饮水工程受益农民不低于10万人；实施五龙背自来水供水项目，保证该地区5000户、1.3万居民饮用水安全；新建一处农副产品交易中心，解决农民卖菜难和市民菜篮子问题；加大检测设备资金投入，建立食品安全检测体系，保障人民群众的食品安全；新建封闭式垃圾压缩中转站，解决垃圾二次造脏污染问题；改造大修20条城市道路；新建农村油路400公里，解决49个行政村通油路问题；开工建设两条城市南北干线，缓解城市交通压力；改造合作区老区，修建2公里沿江景观带；改建七经街和步行街为特色商业街；为城市孤、老、病、残等特殊家庭建设400套廉租房，建筑面积2万平方米；为农村低保户建房2000户，每户补助1.5万元，10月底前全部迁入新居；52个行政村2.2万户实现有线电视“村村通”；所有城市公园7月1日前全部免费开放；实施人民广场美化亮化工程。

切实抓好食品药品安全监管。加快市级食品检测机构食品实验室建设，加强对农产品、食品在生产、加工、流通各环节的安全监管和各级媒体的舆论监督。深入开展食品生产加工企业小作坊、食品批发市场、农村集贸市场等专项整治，重点开展打击违法添加非食用物质和滥用食品添加剂专项整治行动，建立起实现严格监管及食品行业诚信和自律的长效机制。对药品、医疗器械等实施有效的监督和管理。

（十）以繁荣发展各项社会事业为重点，在促进经济社会协调发展上实现新突破

全面协调发展各项社会事业。全面实施素质教育，推进义务教育均衡发展，继续实施新一轮普九规划，加强县级职教中心建设，大力发展职业教育，支持辽东学院等高等院校做大做强。加大对人才和引智工作的投入，重点培养和引进急需的高层次人才。完善城市社区卫生服务体系，全面启动社区卫生服务功能。把所有户籍在农村的农业人口全部纳入新型合作医疗保障体系，参合率达90%以上。科学合理制定新农合补偿方案，扩大提高受益面和受益程度。加强农村三级卫生服务体系建设。积极推进文化信息共享工程进村入户和数字电视整体转换，逐步扩大农村广播电视覆盖面。抓好农村文化服务五项工程，努力满足广大农民文化需求。大力开展全民健身运动，积极发展体育产业。继续稳定低生育水平，提高人口素质。

加强精神文明建设。大力培育和塑造丹东城市精神，进一步宣传丹东城市名片，塑造丹东城市形象。加强公民思想道德建设，抓好“三德”宣传教育。加强诚信建设，倡导社会文明新风尚，创造良好的人文环境和道德支撑。深入开展未成年人思想道德建设，加强青少年社会实践场所的建设和管理，努力营造有利于未成年人健康成长的良好环境。提高城市和农村文明程度、行业执法服务水平，推进社区精神文明建设。

全力维护社会和谐稳定。大力推进平安丹东建设，完善社会治安防控体系，加强边境地区隐蔽战线工作，严厉打击各种违法犯罪活动，确保刑事案件发案率稳中有降。深入开展“五五”普法宣传，不断提高公民的法律素质，切实加强社会治安综合治理和人民调解工作，维护社会的和谐稳定。加强对贫困弱势群体的法律援助，实现法律面前人人平等。建设信访接待大厅，规范信访程序，推进信访工作机制创新，以对人民利益高度负责的态度认真解决群众的合理诉求，深入开展“解难题、促和谐”活动，切实为群众解决实际问题。全面贯彻民族宗教政策，依法管理宗教事务，促进民族团结。深入开展兴边富民行动，加大边境民族地区扶贫开发力度。高度重视安全生产工作，加强对矿山、建筑施工和交通、消防等安全生产的监管整治，继续严厉打击“三无”渔船，坚决杜绝和防止各类重大安全事故的发生。健全突发公共事件应急体系，提高应对突发事件的处理能力。认真开展国防教育，做好民兵预备役工作。

（十一）以提高科学执政能力为重点，在建设人民满意政府上实现新突破

坚持狠抓落实，建设勤政高效政府。全力抓好“落实年”各项工作，重点抓好学习实践科学发展观整改措施的落实，抓好政府既定工作目标任务的落实，抓好项目的落实，抓好转变机关干部作风的落实，确保全市各项工作取得的新起色、新成效。加快政府职能转变和体制创新，强化政府的服务职能，增强领导科学发展、促进社会和谐的能力。深化机构人事制度改革和行政审批制度改革。切实转变工作作风，引导广大机关干部把心思集中在“想干事”上，把精力发挥在“能干事”上，把能力体现在“会干事”上，把目标落实在“干成事”上，努力建设一支高素质的公务员队伍。

坚持依法行政，建设法治民主政府。认真贯彻国务院依法行政实施纲要及加强市县（市）政府依法行政的决定，不断加快依法行政进程。建立健全科学、民主、依法决策机制，严格按照法定权限和程序履行职责。自觉接受人大依法监督和政协民主监督，认真办理建议和提案，继续坚持实行建议、提案办理一把手负责制，提高办理的质量和效率。逐步建立接受民主党派、工商联和无党派人士监督的长效机制。坚持和完善政务公开工作机制，健全重大事项社会公示制度和听证制度，增强政府工作透明度，提高政府公信力。抓好效能监察，继续做好行风政风评议工作，提高行政效能，积极打造促进经济加快发展的软环境，使丹东真正成为亲商、富商、安商之地。

坚持执政为民，建设清正廉洁政府。把为民造福作为政府工作的出发点和落脚点，怀为民之心，思富民之策，行安民之举，以实干的作风取信于民，以发展的成果造福于民。加强政府系统廉政建设，严格执行廉洁从政的各项规定，进一步强化审计监督力度，努力从源头上治理腐败，严厉查处各类违纪违法案件，坚决纠正部门和行业不正之风，真正树立“为民、务实、高效、清廉”的政府形象。

各位代表，2009年我市经济社会发展的任务艰巨而繁重，我们肩负的责任重大而光荣。让我们高举中国特色社会主义伟大旗帜，深入贯彻落实科学发展观，在市委的正确领导下，在市人大、市政协的依法监督与支持下，团结和带领全市人民，进一步解放思想，树立必胜信心，积极应对挑战，齐心协力奋斗，狠抓工作落实，丹东市委、市政府确定的科学发展、和谐发展、跨越发展的目标就一定能如期实现，丹东的明天一定会更加美好。
